# Systematic assessment of the clinicopathological prognostic significance of tissue cytokine expression for lung adenocarcinoma based on integrative analysis of TCGA data

**DOI:** 10.1038/s41598-019-42345-0

**Published:** 2019-04-19

**Authors:** Yuanmei Dong, Yang Liu, Hui Bai, Shunchang Jiao

**Affiliations:** 1 Department of Medical Oncology, Chinese People’s Liberation Army General Hospital, Beijing, 100853 China; 2 The 5th Medical Center, Chinese People’s Liberation Army General Hospital, Beijing, 100039 China; 3 No. 986 Hospital of PLA, Xi’an, 710054 China

**Keywords:** Non-small-cell lung cancer, Tumour biomarkers, Interleukins

## Abstract

Dysregulated intratumoral immune reactions are shaped by complex networks of cytokines, which coordinate with tumor cells to determine tumor progression and aggressiveness. In lung adenocarcinoma (LUAD), the role of intratumoral cytokine gene expression for stratifying prognosis has not been systematically investigated. Using high-dimensional datasets of cancer specimens from clinical patients in The Cancer Genome Atlas (TCGA), we explored the transcript abundance and prognostic impact of 27 clinically evaluable cytokines in 500 LUAD tumor samples according to clinicopathological features and two common driver mutations (EGFR and KRAS). We found that reduced expression of IL12B presented as the single prognostic factor for both poor overall survival (OS) and recurrence free survival (RFS) with high hazard ratios. Moreover, we identified that elevated expression of IL6, CXCL8 and CSF3 were additional independent predictors of poor RFS in LUAD patients. Their prognostic significance was further strengthened by their ability to stratify within clinicopathological factors. Notably, we prioritized high risk cytokines for patients with or without mutations in EGFR and KRAS. Our results provide integrative associations of cytokine gene expression with patient survival and tumor recurrence and demonstrate the necessity and validity of relating clinicopathological and genetic disposition factors for precise and personalized disease prognosis.

## Introduction

Lung cancer is the most common cause of cancer-related death worldwide, with over one million deaths estimated each year. Of note, lung adenocarcinoma (LUAD) represents the most prevalent subtype, and most cases are found at advanced stages. There is an increasing body of epidemiological evidence implicating chronic inflammation in lung cancer etiology^1^, and polymorphisms in inflammation-related genes have been associated with lung cancer risk, particularly LUAD^2,3^. Furthermore, based on the accumulated molecular features of cancer, LUAD has been classified as a complex process that involves a stepwise continuum of tumorigenesis and progression^4^. In this stepwise development, the interplay of host cells and inflammatory cytokines play important roles^5^.

In addition to providing etiologic insight, dysregulated inflammatory cytokines prospectively associated with LUAD could aid in identifying individuals at the highest risk, thereby discovering personalized therapy^6,7^. However, most prior studies are small in size, retrospective in design, and have focused on a few circulating inflammation markers, representing only a small subset of the inflammation cascade^8–10^. Moreover, recent investigations that comprehensively measured a wide range of circulating inflammation markers in relation to lung cancer risk have identified several candidate risk factors that resulted from restrictive conditions^11,12^, failing to provide systematic insight into their clinical prognostic significances.

Launched in 2005, The Cancer Genome Atlas (TCGA) Pilot Project has to date generated clinicopathological annotation data along with multi-platform molecular profiles of more than 11,000 human tumors across 33 different cancer types^13^. Importantly, the currently nested TCGA Pan-Cancer Clinical Data Resource (TCGA-CDR) has collected from 11,600 patients the survival data of four major clinical outcome endpoints, including overall survival (OS), disease-specific survival (DSS), disease-free interval (DFI) and progression-free interval (PFI)^14^. This further strengthens the association between key genomic changes and clinical prognosis, enabling translational studies at both the pan-cancer and individual disease levels. In particular, TCGA Research Network has assessed the molecular profiling (including mutation profiles, structural rearrangements, copy number alterations, DNA methylation, mRNA, miRNA and protein expression) of 585 LUADs to establish a foundation for the classification and further investigations of LUAD molecular pathogenesis. However, a comprehensive analysis to investigate the association of the dysregulation of inflammatory cytokines in tumor tissues with prospective lung cancer risk has not been performed.

In this study, we extracted both genomic and transcriptomic data along with the corresponding clinical pathological information for 500 clinical samples of LUAD from TCGA. We then selected a panel of 27 clinically detectable inflammatory cytokines (Supplementary Table 1), including interleukin-1beta (IL-1β), IL-1ra, IL-2, IL-4, IL-5, IL-6, IL-7, IL-8, IL-9, IL-10, IL-12(p70), IL-13, IL-15, IL-17, eotaxin, fibroblast growth factor-basic (basic FGF), granulocyte colony stimulating factor (G-CSF), granulocyte-macrophage colony stimulating factor (GM-CSF), interferon-gamma (IFN-γ), interferon inducible protein 10 (IP-10), monocyte chemotactic protein 1 (MCP-1), macrophage inflammatory protein 1 alpha (MIP-1α), human platelet derived growth factor BB (PDGF-BB), macrophage inflammatory protein 1 beta (MIP-1β), chemokine (C-C motif) ligand 5 (CCL5), tumor necrosis factor alpha (TNF-α), and vascular endothelial growth factor (VEGF). With regard to clinicopathological features, we focused on major factors, including age, gender, and tumor stage. Notably, using somatic mutation data, the common driver mutation types (EGFR and KRAS) of the selected LUAD patients were also included as important additional factors. Finally, we carried out a systematic assessment to determine the prognostic significance of tissue cytokine gene expression for LUAD patients with respect to survival outcomes of OS and recurrence-free survival (RFS).

## Results

### Clinicopathological statistics of TCGA LUAD Case Subjects

According to TCGA, there are currently 1,089 lung samples, which include 585 LUAD tumor samples. After removing samples with incomplete clinicopathological information, we obtained 500 LUAD tumor samples (refer to Methods). The available clinicopathological information for each sample includes the patient’s age, gender, tumor stage and TNM grouping, together with the gene mutation type. With regard to survival outcome, the survival data provided by UCSC Xena only include OS; thus, we calculated RFS using the data from the phenotypic database.

As shown in Table 1, 54% of the 500 LUAD patients are female and 46% are male, within the age range from 33 to 89. Moreover, only 3.4% of the 500 LUAD patients were diagnosed before the age of 45, whereas most patients (41.6% and 50.8%) were diagnosed between the age of 46 and 65 or older than 65 years. For more than half of LUAD patients (53.6%), tumors were found at stage I, and only 5% of LUAD patients’ tumors were diagnosed at stage IV. Notably, 9.4% of LUAD patients have an EGFR mutation, including 3 G719A/C/S, 19 exon 19 deletions, 2 exon 20 insertions, 2 S768I, 2 T790M, 22 L858R, and 2 L861Q. Moreover, 26% of LUAD patients have a KRAS mutation, and 10 samples have a KRAS copy number amplification. However, only 1.2% of LUAD patients have received EGFR–TKI therapy, leaving the majority of the samples feasible for association analysis.

**Table 1:** Characteristics of lung adenocarcinoma patients in the TCGA Cohort with survival data.

Characteristic	LUAD patient (n = 500)
**Gender**, **n (%)**	
female	270 (54.0)
male	230 (46.0)
**Diagnosis age**, **years**, **n (%)**	
<=45	17 (3.4)
46–65	208 (41.6)
>65	254 (50.8)
Not applicable	21 (4.2)
**Disease stage**	
I	268 (53.6)
II	119 (23.8)
III	80 (16.0)
IV	25 (5.0)
Not applicable	8 (1.6)
**Mutation state**, **n (%)**	
KRAS wildtype	370 (74.0)
KRAS mutant	130 (26.0)
EGFR wildtype	453 (90.6)
EGFR mutant	47 (9.4)
**Received EGFR–TKI therapy**, **n (%)**	
Yes	6 (1.2)
No	494 (98.8)
chemotherapy	160 (32)
other	334 (66.8)

### Association between tissue cytokine expression and survival outcome

**Figure 1:**
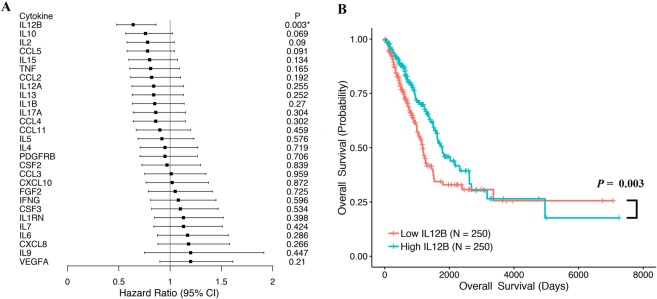
(**A**) Forest plot showing the hazard ratio (HR) and *p* value for overall survival (OS) in patients with lung adenocarcinoma (LUAD) based on cytokine high versus low expression. (**B**) Kaplan-Meier analysis of OS for LUAD patients according to the expression levels of IL12B. LUAD patients were classified into two groups, i.e., patients with high IL12B expression and those with low IL12B expression according to the median IL12B expression. Difference in OS was analyzed with log-rank test.

Using RNA-seq data, the mRNA expression of 27 clinical evaluable cytokines were examined in 500 surgical specimens from TCGA LUAD patients. We calculated the hazard ratio (HR) for OS and RFS in LUAD patients with high versus low tumor cytokine mRNA expression according to the median mRNA expression level in all samples. The association between tissue cytokine mRNA expression and survival outcome was analyzed via Cox regression and Log-rank test and is shown in the forest plots (Fig. 1A; Supplementary Table 2).

Notably, of the 27 cytokines, we only observe statistically significant HR results for OS according to the low mRNA expression level of IL12B in the tumor tissue (Fig. 1A). The Kaplan-Meier curves for LUAD patients according to the expression levels of IL12B are shown in Fig. 1B. Patients with low tumor tissue IL12B mRNA expression had significantly shorter OS than those with high tissue IL12B mRNA expression (HR = 0.64, 95% CI = 0.48–0.86; *p* = 0.003 for high vs. low expression), which demonstrates a shorter median survival time (1171 d for low expression and 1778 d for high expression). IL12B is encoded by two separate genes, IL12B (p40) and IL12A (p35). However, there was no significant HR observed for the IL12A expression in the OS risk evaluation (HR = 0.84, 95% CI = 0.63–1.13, *p* = 0.255 for high vs. low expression).

**Figure 2:**
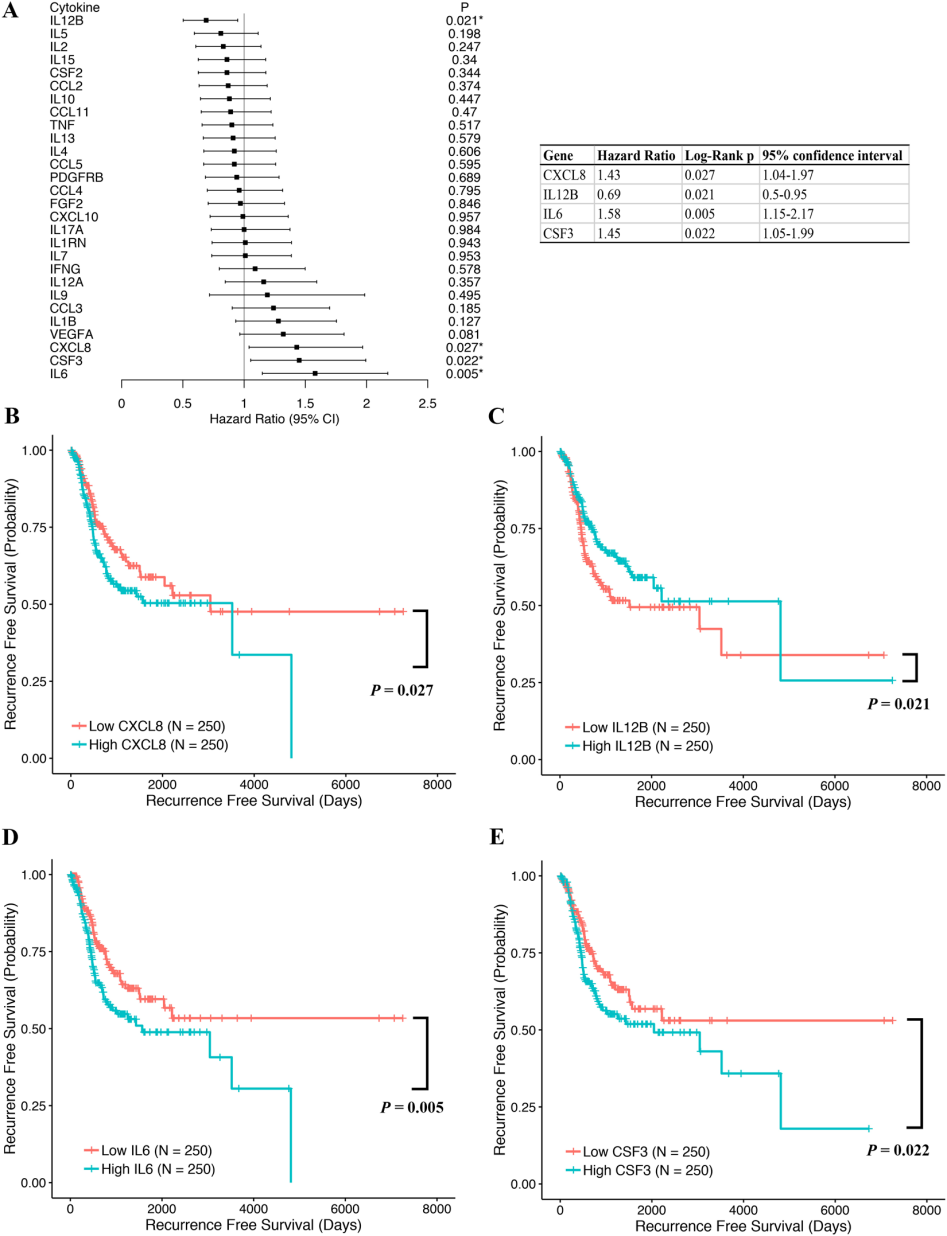
(**A**) Forest plot showing the hazard ratio (HR) and *p* value for recurrence free survival (RFS) in patients with lung adenocarcinoma (LUAD) based on cytokine high versus low expression. Kaplan-Meier analysis of overall survival (OS) for LUAD patients according to the expression levels of (**B**) CXCL8, (**C**) IL12B, (**D**) IL6, and (**E**) CSF3. Patients were classified into two groups, i.e., patients with high cytokine expression and those with low expression according to the median expression. Difference in OS was analyzed with log-rank test.

With respect to RFS, we identified four cytokines with dysregulated expression at the mRNA level that had a significant influence on LUAD patients overall. These cytokine encoding genes included IL12B, IL6, CSF3 (encoding G-CSF), and CXCL8 (encoding IL-8) (Fig. 2A; Supplementary Table 3). The Kaplan-Meier curves for LUAD patients according to the expression levels of CXCL8, IL12B, IL6 and CSF3 are shown in Fig. 2B–E. Patients with low tumor tissue IL12B mRNA expression also had a significantly shorter RFS than those with high IL12B (HR = 0.69, 95% CI = 0.50–0.95, *p* = 0.021 for high vs. low expression). In contrast, the noted elevations of tissue mRNA expression of IL6, CSF3 and CXCL8, respectively, presented in LUAD patients were associated with poor survival before recurrence. Specifically, LUAD patients with higher levels of IL6, CSF3 and CXCL8 showed comparable HRs, i.e., 1.58 (95% CI = 1.15–2.17, *p* = 0.005, for high vs. low IL6 expression), 1.45 (95% CI = 1.05–1.99, *p* = 0.022 for high vs. low CSF3 expression) and 1.43 (95% CI = 1.04–1.97, *p* = 0.027 for high vs. low CXCL8 expression).

As clinicopathological features and mutation types may influence the living risk of LUAD patients, we analyzed the living risks of these 500 LUAD patients according to the previously described parameters and cytokines with univariate and multivariate Cox regression analyses. As shown in Supplementary Table 4, we found that only advanced tumor stages are associated with poor prognosis in both analysis results. Moreover, for the four identified cytokines (IL12B, CXCL8, IL6, and CSF3), only low IL12B expression remains a factor of unfavorable prognosis for both OS and RFS in LUAD patients.

To confirm the models, we searched GEO and validated the results with another cohort (GSE37745^15^), which includes 196 non-small lung cancer (NSCLC) cases (106 LUAD cases) with clinical information and long-term follow-up. Tumor stage (I 70/II 19/III 13/IV 4), age, gender and OS information is available for these data. The original data of all gene expression data for the LUAD sample are normalized with Robust Multi-array Average (RMA^16^) with the newest probe mapping. Univariate and multivariate Cox survival analyses were carried out for the clinicopathological features, including tumor stage, age, gender and gene expression of four cytokines. The results indicate that a low mRNA expression of IL12B is associated with poor prognosis (HR = 0.63, 95% CI = 0.4–1, and p = 0.048 for high vs. low IL12B expression) (Supplementary Table 5).

From these results, we questioned whether the differences in the tumor tissue mRNA expression levels of the 27 cytokines associate with good and poor OS and/or RFS results in LUAD patients with diverse clinicopathological features, including patients’ age at diagnosis, gender and tumor stage. Furthermore, we examined the patient groups with or without EGFR or KRAS mutations showing poor survival. Of note, we placed more focus on IL12B, for which low expression at the mRNA level is prognostic of both poor OS and RFS results.

### Prognostic significance of intratumoral cytokine gene expression for OS in stratified LUAD patient subgroups

**Figure 3:**
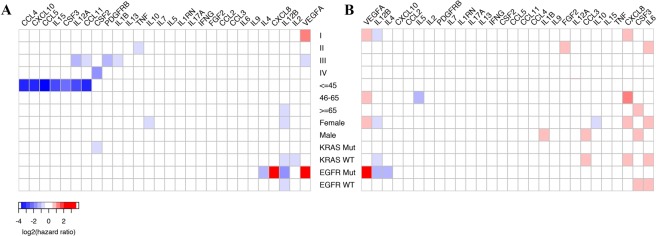
Hazard ratio (HR) heatmap representing associations between cytokine expression and (**A**) overall survival (OS) and (**B**) recurrence free survival (RFS) in different clinicopathological subgroups. The heatmap is colored based on log2 HR. Only HRs with Wald test *p* value less than 0.05 are shown.

To assess the combined survival effect of the tumor tissue mRNA expression of 27 cytokines and variables, such as the clinicopathological parameters and the EGFR/KRAS mutation state, we applied multivariate survival analysis. Figure 3 shows the HRs with statistical significance (Wald test p < 0.05) between high and low cytokine expression under stratification (Supplementary Tables 6 and 7). Poor OS results were clearly observed for most subgroup patients when their tissue expression of specific cytokines was significantly low (blue squares in Fig. 3A).

**Figure 4:**
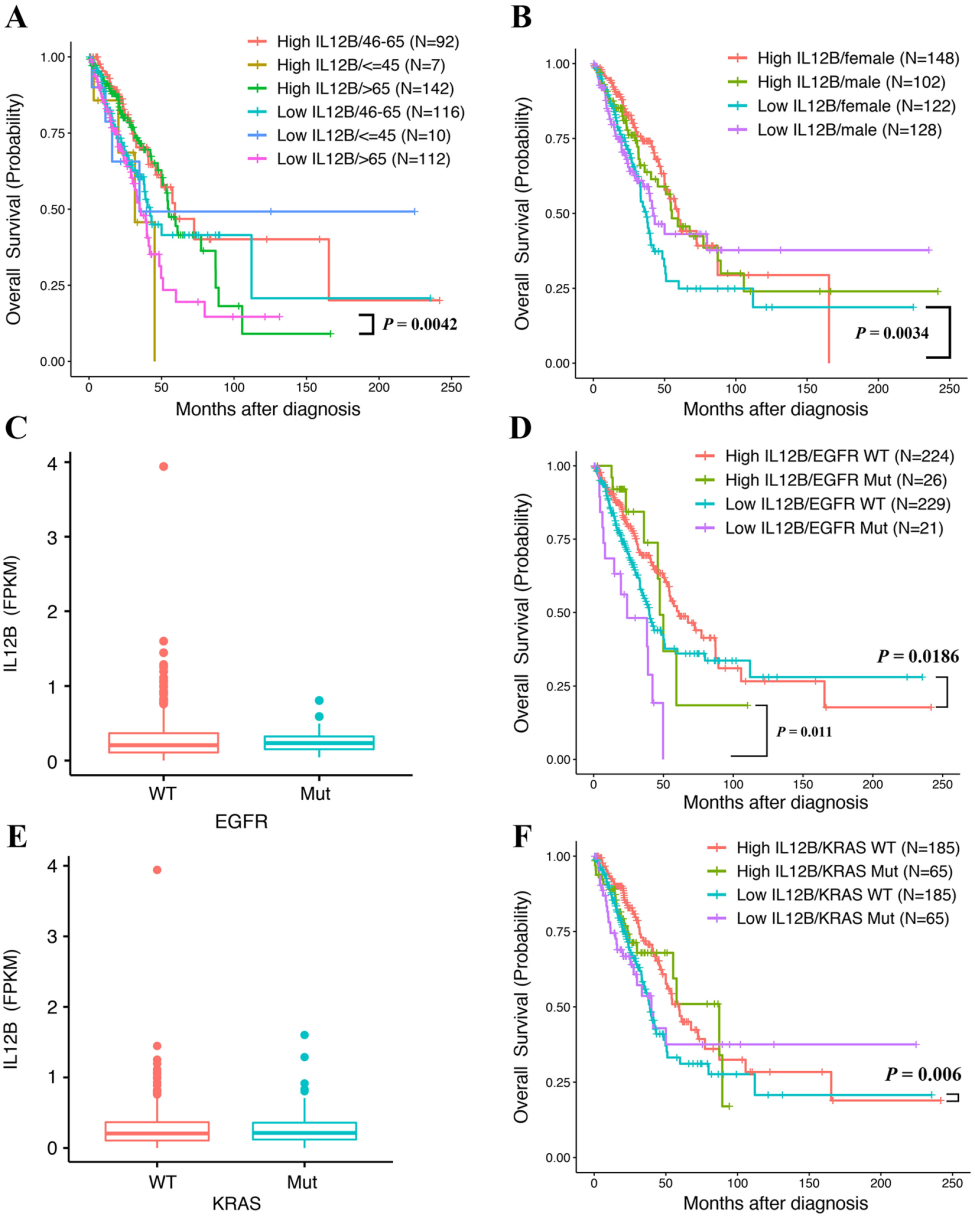
(**A**) Kaplan Meier analysis of overall survival (OS) in six lung adenocarcinoma (LUAD) patient subgroups according to the IL12B expression levels and age at diagnosis. (**B**) Kaplan Meier analysis of OS in four subgroups according to the IL12B expression levels and gender. (**C**) Comparisons of IL12B expression between tumor samples of LUAD patients with or without EGFR mutation. (**D**) Kaplan Meier analysis of OS in four subgroups according to the IL12B expression levels and EGFR mutation status. (**E**) Comparisons of IL12B expression between tumor samples of LUAD patients with or without a KRAS mutation. (**F**) Kaplan Meier analysis of OS in four LUAD patient subgroups according to IL12B expression levels and KRAS mutation status.

First, we observed the prognostic effect of dysregulated cytokines for patients in different diagnosis-age groups. For LUAD patients who were diagnosed >65 years old (n = 254), IL12B was the single cytokine found to predict poor OS (HR = 0.555, 95% CI = 0.371–0.831, and *p* = 0.0042 for the high vs. low IL12B expression >65 subgroup; Fig. 4A), although no significant linear association was observed between patients’ age and expression level of IL12B (Supplementary Fig. 1). However, for LUAD patients diagnosed <45 years old (n = 17), low expression of IL12A (encoding IL-12), CCL5, CCL4 (encoding MIP-1β), CXCL10 (encoding IP-10), CCL11 (encoding Eotaxin), IL15 (encoding IL-15) and CSF3 (encoding G-CSF) was associated with shorter OS, respectively (Fig. 3A). Among these cytokine encoding genes, CCL5 presents the most significant influence on OS, demonstrating a 13.3 times increase in the overall living risk (HR = 0.075, 95% CI = 0.009–0.067, and *p* = 0.016 for the high vs. low CCL5 expression <45 subgroup; Fig. 3A).

Notably, the tissue cytokine expression affects OS of female (n = 270) but not male (n = 230) LUAD patients. Specifically, low IL12B expression was significantly related to the shorter OS of female LUAD patients, increasing their overall living risk by 1.83 (HR = 0.546, 95% CI = 0.364–0.818, and *p* = 0.0034 for the high vs. low IL12B expression female subgroup; Fig. 4B). The other prognostic cytokine for OS of female LUAD patients is IL10, which increased their overall living risk by 1.71 (HR = 0.584, 95% CI = 0.388–0.88, and *p* = 0.0102 for the high vs. low IL10 expression female subgroup; Fig. 3A).

Dysregulated tissue cytokine expression also influences OS of LUAD patients at different tumor stages. For LUAD patients whose tumors were found at the early stage of I, VEGFA was the only prognostic cytokine, and its low tissue mRNA expression level significantly increased their overall living risk by 1.646 (HR = 1.646, 95% CI = 1.01–2.684, and *p* = 0.0456 for the high vs. low VEGFA expression stage I subgroup; Fig. 3A). For stage II LUAD patients, low expression of TNF was prognostic of shorter OS (HR = 0.541, 95% CI = 0.307–0.954, and *p* = 0.0339 for the high vs. low TNF expression stage II subgroup; Fig. 3A). However, for LUAD patients whose tumors were found at the advanced stage IV, low expression of CFS2 (encoding GM-CSF) was prognostic of poor OS (HR = 0.261, 95% CI = 0.084–0.813, and *p* = 0.0205 for the high vs. low CSF2 expression stage IV subgroup; Fig. 3A).

Diverse living risks were observed for LUAD patients with different mutation types if their tumor tissues differentially expressed certain cytokines at the mRNA level. With regard to EGFR wild-type (EGFR^wt^, n = 453) and mutant (EGFR^mut^, n = 47) LUAD patients, no significant difference was observed for the average expression level of IL12B (*p* = 0.39) (Fig. 4C). However, low expression of IL12B in the tumor samples of both the EGFR^wt^ and EGFR^mut^ LUAD patients was related to poor OS, and the overall living risks for EGFR^mut^ LUAD patients was 2 times higher than that observed for the EGFR^wt^ LUAD patients (HR = 0.688, 95% CI = 0.503–0.939, and *p* = 0.0186 for the high vs. low IL12B expression EGFR^wt^ subgroup; HR = 0.318, 95% CI = 0.132–0.769, and *p* = 0.011 for the high vs. low IL12B expression EGFR^mut^ subgroup; Fig. 4D). Notably, IL12B was the only cytokine identified to influence OS of EGFR^wt^ LUAD patients. For EGFR^mut^ LUAD patients, there were three other dysregulated cytokines prognosticating poor OS, including low expressed IL4 (HR = 0.406, 95% CI = 0.172–0.958, and *p* = 0.0395 for the high vs. low IL4 expression EGFR^mut^ subgroup; Fig. 3A) and highly expressed VEGRA (encoding VEGF, HR = 2.79, 95% CI = 1.125–6.919, and *p* = 0.0268 for the high vs. low VEGFA expression subgroup; Fig. 3A) and CXCL8 (HR = 2.585, 95% CI = 1.001–6.677, and *p* = 0.0498 for the high vs. low CXCL8 expression subgroup; Fig. 3A).

As shown in Fig. 4E, no significant difference was observed for the tissue IL12B expression between KRAS wild-type (KRAS^wt^) and mutant (KRAS^mut^) LUAD patients (*p* = 0.64). With similar HRs in KRAS^wt^ LUAD patients (n = 370), IL12B (HR = 0.621, 95% CI = 0.441–0.872, and *p* = 0.006 for the high vs. low IL12B expression KRAS^wt^ subgroup; Fig. 4F) together with IL2 (HR = 0.64, 95% CI = 0.455–0.899, and *p* = 0.0101 for the high vs. low IL2 expression KRAS^wt^ subgroup; Fig. 3A) individually predicted poor OS results when low expressed. However, for KRAS^mut^ LUAD patients (n = 130), CSF2 was the single cytokine encoding gene with low expression related to shorter OS (HR = 0.54, 95% CI = 0.301–0.968, and *p* = 0.0386 for the high vs. low CSF2 expression KRAS^mut^ subgroup; Fig. 3A).

### Prognostic significance of intratumoral cytokine gene expression for RFS in stratified LUAD patient subgroups

RFS has been rated one of the most important endpoints for evaluating cancer progression and the effectiveness of drug treatment. Thus, we attempted to identify dysregulated cytokines that associate with poor RFS for LUAD patients with specific clinicopathological features and mutation types. In general, there are more cytokines with increased than decreased gene expression that affects RFS of LUAD patients stratified in specific subgroups (Fig. 3B).

With respect to patient age at diagnosis, LUAD patients who were diagnosed >65 years old showed a shorter RFS when their tumor expressed a high level of CSF3 (HR = 1.666, 95% CI = 1.09–2.547, and *p* = 0.0184 for the high vs. low CSF3 expression >65 subgroup; Fig. 3B). Regarding the large number of LUAD patients diagnosed between the ages of 46 to 65, abnormal mRNA expression of three cytokines, including CXCL8, VEGFA and IL5, determined their shorter RFS. Specifically, high expression of CXCL8 (HR = 2.059, 95% CI = 1.166–3.634, and *p* = 0.0128 for the high vs. low CXCL8 expression 46–65 subgroup; Fig. 3B) and VEGFA (HR = 1.879, 95% CI = 1.057–3.339, and *p* = 0.0316 for the high vs. low VEGFA expression 46–65 subgroup; Fig. 3B) and low expression of IL5 (HR = 0.478, 95% CI = 0.275–0.833, and *p* = 0.0091 for the high vs. low IL5 expression 46–65 subgroup; Fig. 3B) presented similar risks.

**Figure 5:**
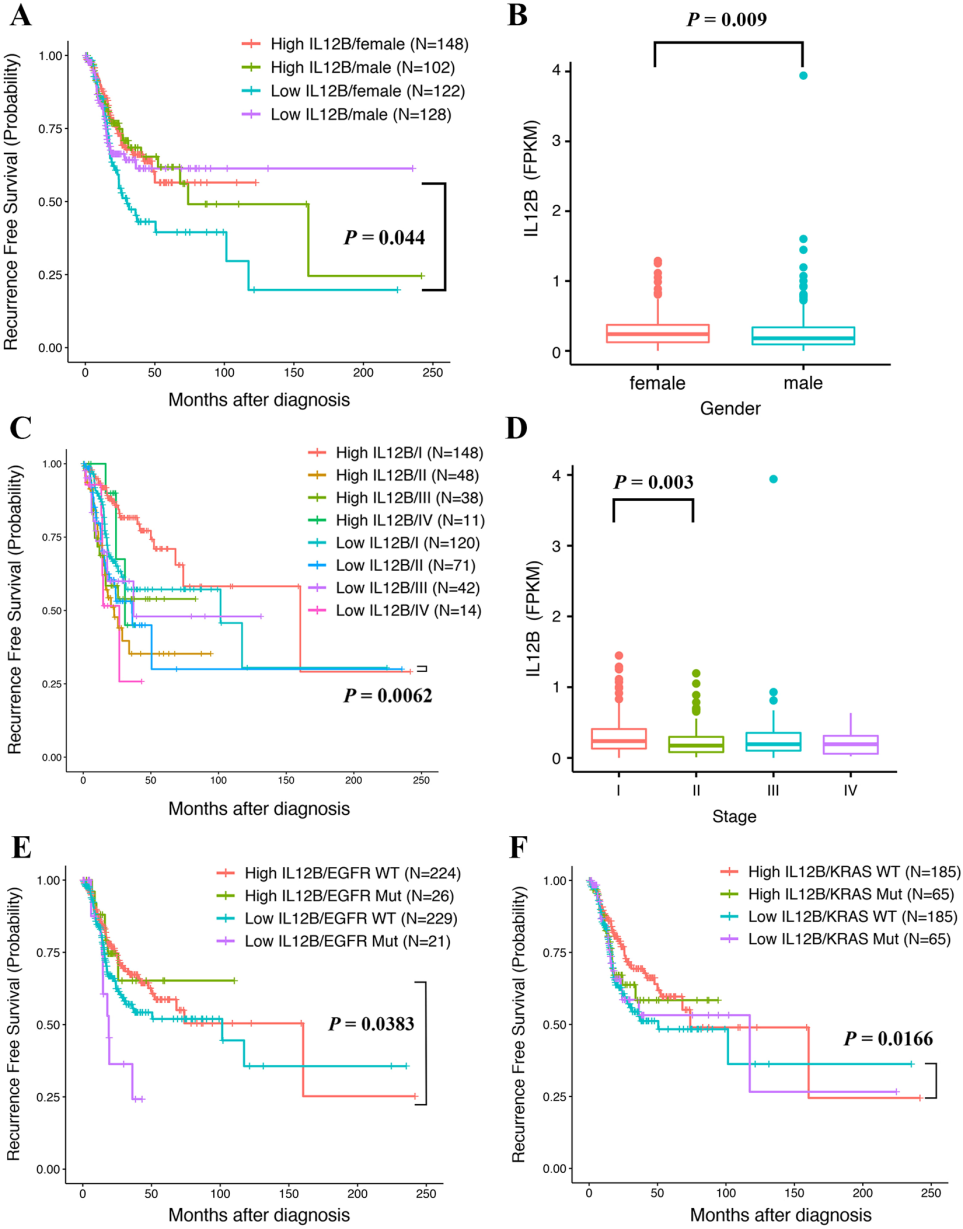
(**A**) Kaplan Meier analysis of recurrence free survival (RFS) in four subgroups according to the IL12B expression levels and gender. (**B**) Comparisons of IL12B expression between tumor samples of lung adenocarcinoma (LUAD) patients with different genders. (**C**) Kaplan Meier analysis of RFS in eight LUAD patient subgroups according to IL12B expression levels and tumor stage. (**D**) Comparisons of IL12B expression between tumor samples of LUAD patients at different pathological stages. (**E**) Kaplan Meier analysis of RFS in four LUAD patient subgroups according to the IL12B expression levels and EGFR mutation status. (**F**) Kaplan Meier analysis of RFS in four LUAD patient subgroups according to the IL12B expression levels and KRAS mutation status.

With respect to gender, there was a distinguished list of cytokines that influenced RFS of female and male LUAD patients. Consistent with the OS results, low expression of IL12B (HR = 0.584, 95% CI = 0.386–0.884, *p* = 0.0109 for the high vs. low IL12B expression female subgroup; Fig. 5A) and IL10 (HR = 0.612, 95% CI = 0.403–0.928, and *p* = 0.0209 for the high vs. low IL10 expression female subgroup; Fig. 3B) significantly increased the living risks of female LUAD patients before tumor recurrence. Moreover, CXCL8, IL6 and VEGFA were the additional cytokine encoding genes that individually influence RFS of female LUAD patients. Their high expression predicted shorter RFS with similar HRs (HR = 1.68, 95% CI = 1.101–2.564, and *p* = 0.0162 for the high vs. low CXCL8 expression female subgroup; HR = 1.54, 95% CI = 1.016–2.334, and *p* = 0.0417 for the high vs. low IL6 expression female subgroup; HR = 1.522, 95% CI = 1.003–2.31, and *p* = 0.0484 for the high vs. low VEGFA expression female subgroup; Fig. 3B). For male LUAD patients, three cytokine encoding genes, including CSF3 (HR = 1.886, 95% CI = 1.136–3.131, and *p* = 0.0142 for the high vs. low CSF3 expression male subgroup; Fig. 3B), CCL3 (encoding MIP-1β, HR = 1.764, 95% CI = 1.071–2.907, and *p* = 0.0259 for the high vs. low CCL3 expression male subgroup; Fig. 3B) and IL1B (encoding IL-1β, HR = 1.659, 95% CI = 1.009–2.726, and *p* = 0.0458 for the high vs. low IL1B expression male subgroup; Fig. 3B), were identified to predict shorter RFS with similar HRs when highly expressed. Notably, a significantly lower average expression level of IL12B was observed for male LUAD patients than that for female LUAD patients (*p* = 0.009, Fig. 5B). However, IL12B showed no such influence on RFS of male LUAD patients (HR = 0.847, 95% CI = 0.517–1.387, and *p* = 0.5094 for the high vs. low IL12B expression male subgroup).

With respect to the tumor stage, we only identified cytokines that significantly influenced RFS of LUAD patients diagnosed at early stages of I (n = 268) and II (n = 119) at mRNA level. Specifically, IL12B showed the most significant influence, and its low expression was prognostic of shorter RFS for stage I LUAD patients (HR = 0.502, 95% CI = 0.306–0.822, and *p* = 0.0062 for the high vs. low IL12B expression stage I subgroup; Fig. 5C). Moreover, there were two cytokine encoding genes with high expression that predicted shorter RFS for stage I LUAD patients with similar HRs, i.e., VEGFA (HR = 1.85, 95% CI = 1.131–3.024, and *p* = 0.0142 for the high vs. low VEGFA expression stage I subgroup, Fig. 3B) and CXCL8 (HR = 1.787, 95% CI = 1.095–2.916, and *p* = 0.0202 for the high vs. low CXCL8 expression stage I subgroup, Fig. 3B). Although a significantly lower average expression level of IL12B was observed for LUAD patients at stage II than I (*p* = 0.003, Fig. 5D), RFS of these patients was not influenced (HR = 1.239, 95% CI = 0.717–2.14, and *p* = 0.4433 for the high vs. low IL12B expression stage II subgroup; Fig. 5C). Of note, high expression levels of FGF2 (encoding Basic FGF, HR = 1.988, 95% CI = 1.15–3.436, and *p* = 0.0138 for the high vs. low FGF2 expression stage II subgroup; Fig. 3B) and IL6 (HR = 1.887, 95% CI = 1.071–3.327, and *p* = 0.028 for the high vs. low IL6 expression stage II subgroup; Fig. 3B) in tumor tissues influenced RFS of stage II LUAD patients.

We have identified distinct dysregulated cytokines affecting the RFS results of EGFR^wt^ and EGFR^mut^ LUAD patients. For EGFR^wt^ LUAD patients (n = 453), IL6 (HR = 1.595, 95% CI = 1.134–2.244, and *p* = 0.0074 for the high vs. low IL6 expression EGFR^wt^ subgroup; Fig. 3B) and CSF3 (HR = 1.51, 95% CI = 1.075–2.12, and *p* = 0.0174 for the high vs. low CSF3 expression EGFR^wt^ subgroup; Fig. 3B) predicted poor RFS results with similar HRs when highly expressed. For EGFR^mut^ LUAD patients (n = 47), low expression of IL12B (HR = 0.36, 95% CI = 0.137–0.947, and *p* = 0.0383 for the high vs. low IL12B expression EGFR^mut^ subgroup; Fig. 5E) together with IL4 (HR = 0.382, 95% CI = 0.147–0.991, and *p* = 0.0479 for the high vs. low IL4 expression EGFR^mut^ subgroup; Fig. 3B) were individually prognostic of a shorter RFS, increasing living risks before tumor recurrence by 2.78 and 2.62 times, respectively. Notably, EGFR^mut^ LUAD patients with a high expression level of VEGRA presented the highest living risks (HR = 4.96, 95% CI = 1.616–15.222, and *p* = 0.0051 for the high vs. low VEGFA expression EGFR^mut^ subgroup; Fig. 3B).

With regard to KRAS^wt^ LUAD patients, shorter RFS was observed when three individual cytokines were highly expressed, including IL6 (HR = 1.539, 95% CI = 1.006–2.221, and *p* = 0.0213 for the high vs. low IL6 expression KRAS^wt^ subgroup; Fig. 3B), CCL3 (HR = 1.541, 95% CI = 1.062–2.237, and *p* = 0.023 for the high vs. low CCL3 expression KRAS^wt^ subgroup; Fig. 3B) and CXCL8 (HR = 1.473, 95% CI = 1.02–2.217, and *p* = 0.0387 for the high vs. low CXCL8 expression KRAS^wt^ subgroup; Fig. 3B). Moreover, IL12B was the single prognostic cytokine with low expression that led to shorter RFS of KRAS^wt^ LUAD patients, increasing living risks before tumor recurrence by 1.57 times (HR = 0.638, 95% CI = 0.442–0.921, and *p* = 0.0166 for the high vs. low IL12B expression KRAS^wt^ subgroup, Fig. 5F). Unfortunately, no cytokine with dysregulated gene expression at the mRNA level was found to be significantly related to poor RFS results for KRAS^mut^ LUAD patients.

## Discussion

Several studies of primary LUAD have reported the ability to examine tissue cytokine expression levels that can be used to group subjects according to their survival outcomes^10,17^. However, most studies are small (approximately 100 subjects or less) and typically drew data from a single treatment institution. The published cytokines often show little overlap in the encoding genes identified as significant predictors of outcome. To date, TCGA has provided the largest publicly recognizable tumor tissue RNA-seq data with real clinical applicability plus variability in samples collected from different institutions. Limited data have been published on the prognostic significance of cytokines in tumor tissues of LUAD for performing analyses in larger data sets and refined stratification. Thus, we systematically assessed the mRNA expression levels of 28 cytokine encoding genes in TCGA tumor samples of 500 LUAD patients according to diverse clinicopathological factors (age, gender and tumor stage) and common driver mutations (EGFR and KRAS) and thereby determined their prognostic values with respect to overall survival and time to recurrence.

For all LUAD patients regardless of the subgroup, we found that reduced expression of IL12B at the mRNA level is the single prognostic factor for both poor OS and RFS results. IL-12 is a known proinflammatory cytokine that mediates both adaptive and innate immunity by targeting multiple immune cells^18^. As an upstream immunomodulator, it exerts potent anti-tumor activity through a combination of immunostimulatory and anti-angiogenic mechanisms. The latter are related to the induction of IFN-γ, which, in turn, triggers the release of the anti-angiogenic chemokines CXCL9, CXCL10 and CXCL11 and the down-regulation of the production of the pro-angiogenic molecules VEGF and FGF-2^19^. Thus, the quest for using this potent cytokine for the immunotherapy of cancer has continued for 20 years^20,21^. With regard to LUAD, the pivotal role of IL-12 and its receptor IL-12Rβ2 has been confirmed in many studies. In the early study of Pistoia *et al*., the administration of IL-12 directly inhibited the growth of human IL-12R^+^ LUAD cells *in vitro* and *in vivo*^22^. Lv *et al*. have achieved stable expression of IL-12 in adipose-derived mesenchymal stem cells through transgenic technology and utilize the paracrine effect of IL-12 to inhibit LUAD cell migration and invasion^23^. In addition, a lack of IL-12 signaling in il12rb2 knock-out mice was found to predispose to LUAD^24^, and a higher frequency of il12rb2 polymorphisms was significantly associated with an increased probability of LUAD occurrence^25^. In the immune microenvironment, the tumor expression level of IL-12Rβ2 was proven to be an independent predictor of recurrence in patients with stage I LUAD^26^.

In the present study, we separately evaluated the tumor expression level of the IL-12 encoding genes IL12B and IL12A. Our multivariate analysis elucidated that low expression of IL12A was only associated with an unfavorable OS prognosis in LUAD patients <45 years old or at stage III, the number of which is relatively small and thus merits further investigation. Importantly, the prognostic significance of IL12B was further strengthened by its ability to stratify within many currently known prognosticators, including gender (female), age at diagnosis (>65 for OS), tumor stage I, with EGFR mutation, and without KRAS mutation. Taken together, IL12B is a stronger predictor of survival outcome for LUAD patients. Our results provide new support for the applicability of IL12B as an immune marker for predicting living risk in a specific population.

Increasing evidence has indicated an increasing rate of tumor recurrence in LUAD patients; therefore, the prognosis of recurrence with regard to the immune component may provide valuable information for better comprehension of the progression of the disease and curative options. Our analysis suggested that elevated expression of IL6, CXCL8 and CSF3 constituted additional independent predictors of poor RFS, presenting similar risks in the same patient subgroups of IL12B. The mechanisms that underlie IL-6 increased metastasis and the effect of IL-6 treatment in lung cancer have been demonstrated *in vitro* and *in vivo*^27–30^ and similar results have been obtained for CXCL8^31,32^. Furthermore, their prognostic significance was demonstrated via associations with unique clinicopathological factor(s). Specifically, IL-6 individually associates with an unfavorable prognosis in LUAD patients between the age of 46 and 65 or tumors at stage II; female LUAD patients and those with tumors diagnosed at stage I or without KRAS mutation suffer a higher risk of tumor occurrence if their tumors highly express CXCL8; for male LUAD patients, this is also the case if their tumors highly express CSF3. Altogether, these results contribute new evidence to the necessity of stratification and thus personalized prognosis based on cytokine gene expression.

Another notable advantage of our systematic assessment is that common driver mutation genes of EGFR and KRAS were included as stratification factors. For the 453 (90.6%) EGFR^wt^ LUAD patients in our analysis, IL12B was the single predictor for OS. Although the number of EGFR^mut^ LUAD patients was relatively small (n = 47, 9.4%), a high level of intratumoral VEGFA was identified to prognosticate the highest risk in RFS. Lord and his colleagues have proven in a mouse model that intratumoral IL-12 resulted in a significant delay in tumor growth and phenotypic changes in the vasculature, and vessels within B16/IL-12 tumors have a more normal morphology and do not express VEGFR3^33^. Similarly, Heymach *et al*. have established anti-VEGFR-2 and IL-12-engineered T cells to induce the regression of 5 different vascularized syngeneic tumors without the addition of exogenous IL-2 in mouse models^34^. Importantly, Heymach *et al*. have reported that patients with non-small-cell lung cancer benefiting longer RFS from Vandetanib (anti-EGFR and anti-VEGRR2) monotherapy or in combination with chemotherapy result from the plasma IL-12 level not reduced after treatment^35^. Collectively, these reports further reflected the regulatory relation between IL-12 and VEGFR and thus the importance of the IL-12 expression level in anti-VEGFR therapy for cancer patients.

Mutations in the proto-oncogene KRAS occur in 30% to 50% of LUAD. Unfortunately, attempts to target KRAS mutant lung tumors have thus far failed. For the 130 (26%) KRAS^mut^ LUAD patients included in our analysis, we have identified only one cytokine gene CSF2 with a significant HR in the OS risk evaluation. Although lacking evidence from LUAD, several studies have suggested that aberrant KRAS signaling is responsible for triggering immunological responses and inflammation-driven tumorigenesis, in which a marked induction of CSF2 expression has been observed in an ovarian cancer cell line^36^ and colorectal cancer patients with progression^37^. The ability to prognosticate within tumors without the KRAS mutation is of particular importance because this group represents a population that cannot benefit from targeted therapy and in which the efficacy is quite limited under the treatment of standard chemotherapy. For 370 (74%) KRAS^wt^ LUAD patients in our analysis, low expression of IL12B predicted worse OS and RFS, while patients with high expression of IL6, CCL3 and CXCL8 experienced similar risks in the RFS evaluation. Moghaddam *et al*. have utilized a K-ras induced lung cancer mouse model to demonstrate that K-ras activation drives a pro-tumor immune suppressive microenvironment with increased type 2 and reduced type 1 inflammatory signatures via IL-6 signaling. Significantly, they show that pharmacologic targeting of IL-6 suppresses K-ras lung tumorigenesis and re-educates the lung microenvironment toward an anti-tumor immune phenotype^38^. Bar-Sagi *et al*. have showed that CXCL8 is a transcriptional target of Ras signaling and is required for the initiation of tumor-associated inflammation and neovascularization in xenograft models^39^.

In summary, we link concentrations of intratumoral cytokines to patient survival and tumor recurrence. We highlight the necessity and validity of relating clinicopathological and genetic disposition factors to the prioritization of highly risky cytokines and their integrative impacts on precise and personalized disease prognosis. The results from this systematic analysis would create clinical benefits if more LUAD patients in special subgroups are included.

## Methods

### Data acquisition

The clinicopathological information and genomic and transcriptomic profiles of LUAD patients in TCGA were downloaded from UCSC Xena, a platform that provides multiple source normalized datasets, including TCGA. Only tumor samples with fully transcriptomic profile and survival data were included, resulting in 500 samples. The clinicopathological information includes the following metrics: pathological stage, age, gender, OS, vital status, new tumor event after initial treatment and days to new tumor event after initial treatment. RFS was calculated based on the new tumor event after initial treatment and days to the new tumor event after initial treatment. For RNA-seq data, Fragments Per Kilobase of transcript per Million mapped reads (FPKM) was used as a unit representing the expression levels of genes. Somatic mutations, such as SNP and INDELs, were from MuTect2^40^.

### Sample grouping

For cytokine expression, samples were categorized into high and low expression groups based on the median expression (FPKM) of the specific cytokine across all samples^41^. Age was divided into three groups, i.e., <=45, 46–65 and >65. For KRAS and EGFR, samples were categorized as mutant and wild groups based on the mutation status of EGFR (including G719A/C/S, exon 19 deletions, exon 20 insertions, S768I, T790M, L858R, and L861Q)^42^ and KRAS (including mutations at the 12th, 13th, and 61st codons)^43^.

### Statistical analysis

The association between cytokine expression levels and survival (OS and RFS) was carried out by univariate Cox regression. The Log-rank test was used to compare the significance of the high and low expression groups. The association between different clinical parameters and survival was performed via univariate Cox regression and multivariate Cox regression analyses. The Kaplan Meier survival plot was generated using R/survminer package^44^. The cytokine expression differences between subgroups of the clinicopathological parameters were examined using the Mann–Whitney test (variables with two levels, such as the KRAS status) or the Kruskal–Wallis test (variables with multiple levels, such as the clinical stage). To assess the combined survival effect of cytokine expression under different clinical parameter subgroups, i.e., clinical stage, age, or gender, multivariate survival analysis was performed^45^. Moreover, the Wald test was employed to assess the statistical significance of the survival correlation between high and low cytokine expression for a given subgroup. Analyses were performed using R version 3.5.0^46^ and SPSS version 25.

## Supplementary information

Supplementary materials

## Data Availability

The datasets generated and/or analyzed during the current study are available from the corresponding author on reasonable request.
