# Supplementary material for: Systematic assessment of the clinicopathological prognostic significance of tissue cytokine expression for lung adenocarcinoma based on integrative analysis of TCGA data
Source: Sci Rep. 2019 Apr 19;9:6301. doi: 10.1038/s41598-019-42345-0 (PMC6474906; doi:10.1038/s41598-019-42345-0)
Supplement: Supplementary file 1 — Supplementary materials [file 41598_2019_42345_MOESM1_ESM.docx]

Systematic assessment of the clinicopathological prognostic significance of tissue cytokine expression for lung adenocarcinoma based on integrative analysis of TCGA data

Yuanmei Dong ^1,^*, Yang Liu ^2,^*, Hui Bai ^3,^†, Shunchang Jiao ^1,^†

1. Department of Medical Oncology, Chinese People’s Liberation Army General Hospital, Beijing 100853, China.
2. The 5th Medical Center, Chinese People’s Liberation Army General Hospital, Beijing 100039, China., China.
3. No. 986 Hospital of PLA, Xi’an 710054, China.

***** These authors contributed equally to this work.

**†** Correspondence: Shunchang Jiao, jiaosc06@sina.com, Tel.: +86-010-66937002; Hui Bai, huibai13@hotmail.com, Tel.: +86-010-66932251.


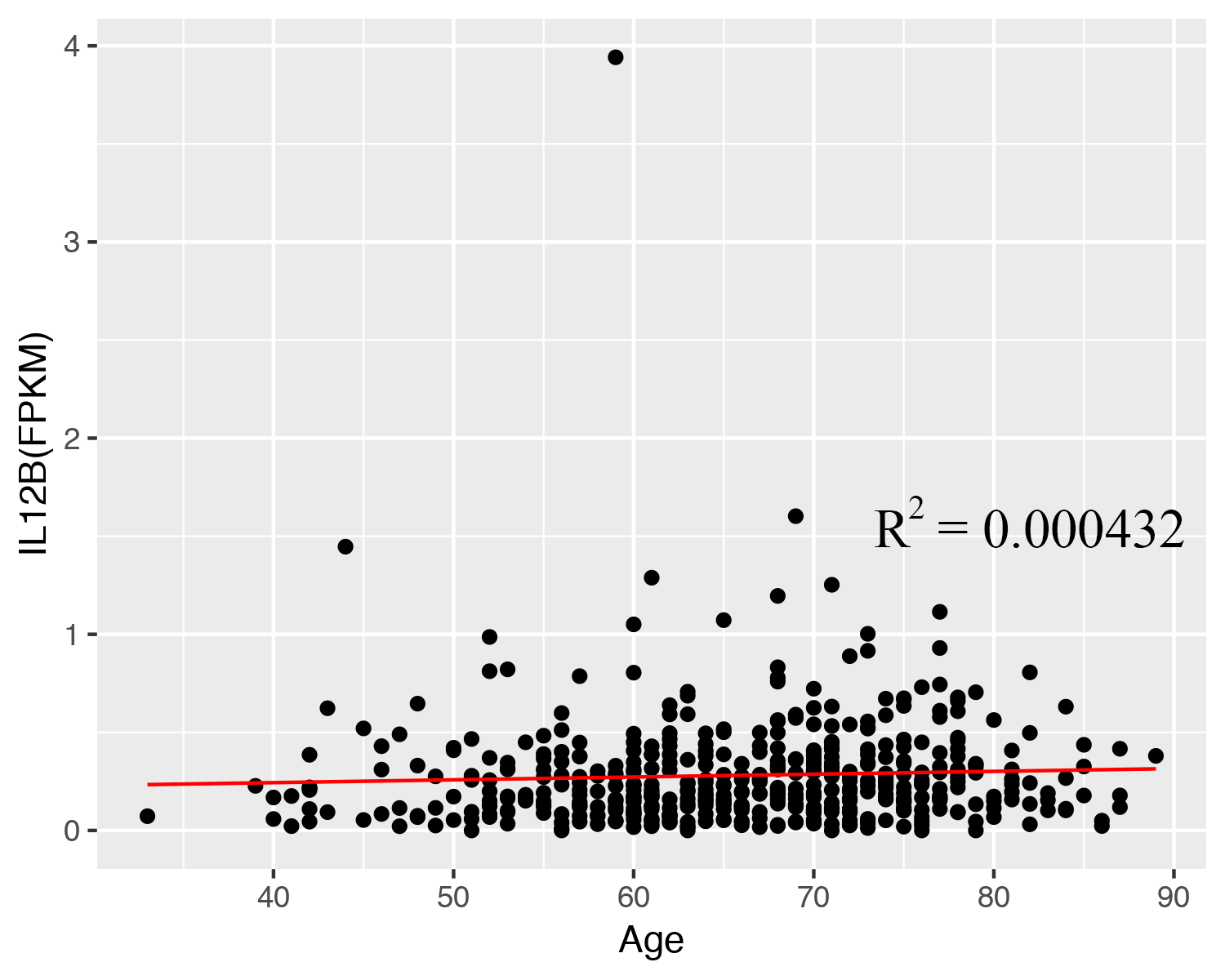


**Supplementary Fig. 1**. **Correlations between IL12B expression and diagnosis age in lung adenocarcinomas.** The red line is the linear regression of IL12B expression on age. The adjusted coefficient of determination (R-squared) is 0.000432, which means age accounts for none of the variation in IL12B expression.

**Supplemental Table 1.** 27 clinically detectable inflammatory cytokines used in this study.

| **Cytokine** | **Gene** |
| --- | --- |
| IL-1beta | IL1B |
| IL-1ra | IL1RN |
| IL-2 | IL2 |
| IL-4 | IL4 |
| IL-5 | IL5 |
| IL-6 | IL6 |
| IL-7 | IL7 |
| IL-8 | CXCL8 |
| IL-9 | IL9 |
| IL-10 | IL10 |
| IL-12(p70) | IL12A/IL12B |
| IL-13 | IL13 |
| IL-15 | IL15 |
| IL-17 | IL17A |
| Eotaxin | CCL11 |
| Basic FGF | FGF2 |
| G-CSF | CSF3 |
| GM-CSF | CSF2 |
| IFN-gamma | IFNG |
| IP-10 | CXCL10 |
| MCP-1 | CCL2 |
| MIP-1 alpha | CCL3 |
| PDGF-BB | PDGFRB |
| MIP-1beta | CCL4 |
| RANTES | CCL5 |
| TNF-alpha | TNF |
| VEGF | VEGFA |

**Supplemental Table 2.** Survival analysis results for OS in LUAD patients with high versus low cytokine mRNA expression.

| **Gene** | **Hazard Ratio** | **Log-Rank p** | **95% CI** | **Median survival(low-high)** |
| --- | --- | --- | --- | --- |
| IL10 | 0.76 | 0.069 | 0.57-1.02 | 1235-1531 |
| IL1B | 0.85 | 0.27 | 0.63-1.14 | 1379-1653 |
| IL1RN | 1.13 | 0.398 | 0.85-1.52 | 1516-1357 |
| IL12A | 0.84 | 0.255 | 0.63-1.13 | 1268-1528 |
| CXCL8 | 1.18 | 0.266 | 0.88-1.58 | 1531-1421 |
| CXCL10 | 1.02 | 0.872 | 0.76-1.37 | 1501-1498 |
| IL2 | 0.78 | 0.09 | 0.58-1.04 | 1215-1778 |
| FGF2 | 1.05 | 0.725 | 0.79-1.41 | 1516-1421 |
| IL15 | 0.8 | 0.134 | 0.6-1.07 | 1268-1531 |
| CSF2 | 0.97 | 0.839 | 0.73-1.3 | 1258-1528 |
| IL5 | 0.92 | 0.576 | 0.69-1.23 | 1421-1501 |
| IL13 | 0.84 | 0.252 | 0.63-1.13 | 1265-1653 |
| IL4 | 0.95 | 0.719 | 0.71-1.27 | 1498-1492 |
| IL9 | 1.2 | 0.447 | 0.75-1.91 | 1501-1147 |
| PDGFRB | 0.95 | 0.706 | 0.71-1.27 | 1492-1516 |
| IL12B | 0.64 | 0.003 | 0.48-0.86 | 1171-1778 |
| TNF | 0.81 | 0.165 | 0.61-1.09 | 1258-1600 |
| VEGFA | 1.2 | 0.21 | 0.9-1.61 | 1499-1498 |
| IL17A | 0.86 | 0.304 | 0.64-1.15 | 1357-1600 |
| IL6 | 1.17 | 0.286 | 0.88-1.57 | 1501-1357 |
| IL7 | 1.13 | 0.424 | 0.84-1.51 | 1501-1492 |
| IFNG | 1.08 | 0.596 | 0.81-1.45 | 1492-1499 |
| CCL2 | 0.82 | 0.192 | 0.62-1.1 | 1454-1499 |
| CCL11 | 0.9 | 0.459 | 0.67-1.2 | 1454-1499 |
| CCL5 | 0.78 | 0.091 | 0.58-1.04 | 1379-1531 |
| CCL3 | 1.01 | 0.959 | 0.75-1.35 | 1501-1498 |
| CCL4 | 0.86 | 0.302 | 0.64-1.15 | 1379-1600 |
| CSF3 | 1.1 | 0.534 | 0.82-1.47 | 1499-1498 |

**Supplemental Table 3.** Survival analysis result for RFS in LUAD patients with high versus low cytokine mRNA expression.

| **Gene** | **Hazard Ratio** | **Log-Rank p** | **95% CI** | **Median survival(low-high)** |
| --- | --- | --- | --- | --- |
| IL10 | 0.88 | 0.447 | 0.64-1.21 | 2218-3521 |
| IL1B | 1.28 | 0.127 | 0.93-1.75 | 3044-3521 |
| IL1RN | 1.01 | 0.943 | 0.74-1.39 | 3044-3521 |
| IL12A | 1.16 | 0.357 | 0.85-1.59 | NA-2045 |
| CXCL8 | 1.43 | 0.027 | 1.04-1.97 | 3044-3521 |
| CXCL10 | 0.99 | 0.957 | 0.72-1.36 | 2218-3521 |
| IL2 | 0.83 | 0.247 | 0.61-1.14 | 2045-3044 |
| FGF2 | 0.97 | 0.846 | 0.71-1.33 | 3044-2218 |
| IL15 | 0.86 | 0.34 | 0.63-1.18 | 2218-3521 |
| CSF2 | 0.86 | 0.344 | 0.63-1.18 | 3044-3521 |
| IL5 | 0.81 | 0.198 | 0.59-1.11 | 3044-4812 |
| IL13 | 0.91 | 0.579 | 0.67-1.25 | 1568-3044 |
| IL4 | 0.92 | 0.606 | 0.67-1.26 | 2218-3044 |
| IL9 | 1.19 | 0.495 | 0.72-1.99 | 3044-2218 |
| PDGFRB | 0.94 | 0.689 | 0.68-1.29 | 2045-3521 |
| IL12B | 0.69 | 0.021 | 0.5-0.95 | 1516-4812 |
| TNF | 0.9 | 0.517 | 0.66-1.24 | 2218-3521 |
| VEGFA | 1.32 | 0.081 | 0.96-1.82 | 4812-3044 |
| IL17A | 1 | 0.984 | 0.73-1.37 | 3044-2218 |
| IL6 | 1.58 | 0.005 | 1.15-2.17 | NA-1568 |
| IL7 | 1.01 | 0.953 | 0.74-1.39 | 3044-1568 |
| IFNG | 1.09 | 0.578 | 0.8-1.5 | 3044-3521 |
| CCL2 | 0.87 | 0.374 | 0.63-1.19 | 2218-3521 |
| CCL11 | 0.89 | 0.47 | 0.65-1.22 | 2045-3521 |
| CCL5 | 0.92 | 0.595 | 0.67-1.26 | 3044-3521 |
| CCL3 | 1.24 | 0.185 | 0.9-1.7 | 4812-3044 |
| CCL4 | 0.96 | 0.795 | 0.7-1.31 | 2218-3521 |
| CSF3 | 1.45 | 0.022 | 1.05-1.99 | NA-2045 |

**Supplemental Table 4.** Univariate and multivariate Cox regression analysis for survival in 500 LUAD patients with clinicopathological features and cytokine mRNA expression.

| **Variable** | **Univariate analysis** | | | | **Multivariate analysis** | | | |
| --- | --- | --- | --- | --- | --- | --- | --- | --- |
|  | **Hazard Ratio** | **95% CI** | **P-value** | **Hazard Ratio** | | **95% CI** | **P-value** |  |
| **OS** |  |  |  |  | |  |  |  |
| gender (male vs. female) | 1.05 | 0.78-1.4 | 0.75 | 0.95 | | 0.7-1.29 | 0.74 |  |
| KRAS_SNV (Mut vs. Wild) | 1.1 | 0.79-1.54 | 0.57 | 1.17 | | 0.83-1.66 | 0.37 |  |
| EGFR_SNV (Mut vs. Wild) | 1.49 | 0.94-2.34 | 0.087 | 1.35 | | 0.83-2.18 | 0.22 |  |
| stage (II vs. I) | 2.47 | 1.72-3.56 | **6.61E-07** | 2.41 | | 1.66-3.51 | **4.27E-06** |  |
| stage (III vs. I) | 3.5 | 2.38-5.13 | **5.44E-11** | 3.34 | | 2.26-4.93 | **1.24E-09** |  |
| stage (IV vs. I) | 3.82 | 2.2-6.63 | **3.23E-07** | 3.84 | | 2.17-6.78 | **3.67E-06** |  |
| T (T2 vs. T1) | 1.45 | 1.02-2.07 | **0.046** |  | |  |  |  |
| T (T3 vs. T1) | 2.97 | 1.77-5 | **0.0002** |  | |  |  |  |
| T (T4 vs. T1) | 2.91 | 1.5-5.66 | **0.003** |  | |  |  |  |
| N (N1 vs. N0) | 2.39 | 1.69-3.37 | **4.55E-07** |  | |  |  |  |
| N (N2/N3 vs. N0) | 2.9 | 1.98-4.25 | **4.47E-08** |  | |  |  |  |
| M (M1 vs. M0) | 2.13 | 3.65 | **0.0047** |  | |  |  |  |
| Age (46-65 vs. <=45) | 0.79 | 0.38-1.64 | 0.52 | 0.76 | | 0.35-1.65 | 0.49 |  |
| Age (>65 vs. <=45) | 0.96 | 0.46-1.99 | 0.91 | 1.04 | | 0.49-2.24 | 0.91 |  |
| IL12B (High vs. Low) | 0.64 | 0.48-0.86 | **0.003** | 0.66 | | 0.49-0.89 | **0.006** |  |
| **RFS** |  |  |  |  | |  |  |  |
| gender (male vs. female) | 0.87 | 0.63-1.2 | 0.4 | 0.96 | | 0.7-1.3 | 0.77 |  |
| KRAS_SNV (Mut vs. Wild) | 1.11 | 0.78-1.6 | 0.56 | 1.19 | | 0.84-1.69 | 0.33 |  |
| EGFR_SNV (Mut vs. Wild) | 1.29 | 0.78-2.14 | 0.32 | 1.49 | | 0.91-2.46 | 0.11 |  |
| stage (II vs. I) | 2.35 | 1.63-3.4 | **4.52E-06** | 2.4 | | 1.65-3.51 | **5.50E-06** |  |
| stage (III vs. I) | 2.02 | 1.29-3.14 | **0.002** | 3.4 | | 2.28-5.06 | **1.80E-09** |  |
| stage (IV vs. I) | 1.91 | 0.95-3.84 | 0.07 | 3.98 | | 2.24-7.06 | **2.32E-06** |  |
| T (T2 vs. T1) | 1.71 | 1.17-2.49 | **0.005** |  | |  |  |  |
| T (T3 vs. T1) | 2.89 | 1.64-5.08 | **0.0002** |  | |  |  |  |
| T (T4 vs. T1) | 1.24 | 0.44-3.49 | 0.68 |  | |  |  |  |
| N (N1 vs. N0) | 1.75 | 1.2-2.54 | **0.004** |  | |  |  |  |
| N (N2/N3 vs. N0) | 1.81 | 1.18-2.8 | **0.007** |  | |  |  |  |
| M (M1 vs. M0) | 1.41 | 0.71-2.79 | 0.33 |  | |  |  |  |
| Age (46-65 vs. <=45) | 0.81 | 0.35-1.9 | 0.63 | 0.83 | | 0.38-1.81 | 0.64 |  |
| Age (>65 vs. <=45) | 1.18 | 0.51-2.73 | 0.7 | 1.12 | | 0.51-2.43 | 0.78 |  |
| IL12B (High vs. Low) | 0.69 | 0.5-0.95 | **0.021** | 0.65 | | 0.48-0.88 | **0.005** |  |
| CXCL8 (High vs. Low) | 1.43 | 1.04-1.97 | **0.027** | 0.89 | | 0.63-1.26 | 0.5 |  |
| IL6 (High vs. Low) | 1.58 | 1.15-2.17 | **0.005** | 1.33 | | 0.93-1.9 | 0.12 |  |
| CSF3 (High vs. Low) | 1.45 | 1.05-1.99 | **0.022** | 1.01 | | 0.73-1.38 | 0.97 |  |

**Supplemental Table 5.** Univariate and multivariate Cox regression analysis for survival in LUAD patients (GSE37745) with clinicopathological features and cytokine mRNA expression.

| **Variable** | **Univariate analysis** | | | **Multivariate analysis** | | |
| --- | --- | --- | --- | --- | --- | --- |
|  | **Hazard Ratio** | **95% CI** | **P-value** | **Hazard Ratio** | **95% CI** | **P-value** |
| stage (II vs. I) | 1.47 | 0.83-2.6 | 0.18 | 1.51 | 0.83-2.78 | 0.18 |
| stage (III vs. I) | 2.1 | 1.11-3.99 | **0.02** | 1.8 | 0.91-3.56 | 0.09 |
| stage (IV vs. I) | 1.53 | 0.47-4.93 | 0.48 | 1.59 | 0.47-5.43 | 0.46 |
| age (>65 vs. <=65) | 1.47 | 0.94-2.3 | 0.09 | 1.24 | 0.77-2.01 | 0.37 |
| gender (male vs. female) | 1.26 | 0.8-1.97 | 0.32 | 1.34 | 0.84-2.14 | 0.22 |
| IL12B (High vs. Low) | 0.63 | 0.4-1 | **0.048** | 0.64 | 0.39-1.06 | 0.085 |
| CXCL8 (High vs. Low) | 0.97 | 0.62-1.52 | 0.90 | 0.88 | 0.51-1.52 | 0.66 |
| IL6 (High vs. Low) | 0.98 | 0.62-1.52 | 0.91 | 0.99 | 0.56-1.75 | 0.96 |
| CSF3 (High vs. Low) | 0.84 | 0.53-1.31 | 0.43 | 0.77 | 0.47-1.27 | 0.31 |

**Supplemental Table 6.** Survival analysis result for OS in LUAD patients with high and low cytokine expression under stratification of clinicopathological parameters.

| **Cytokine** | **Clinicopathological parameters** | **Group** | **Hazard Ratio** | **95% CI** | **Wald Test P** |
| --- | --- | --- | --- | --- | --- |
| IL1B | stage | III | 0.52 | 0.281-0.961 | 0.0369 |
| IL12A | stage | III | 0.437 | 0.241-0.795 | 0.0067 |
| CSF2 | stage | IV | 0.261 | 0.084-0.813 | 0.0205 |
| PDGFRB | stage | III | 0.45 | 0.248-0.817 | 0.0086 |
| TNF | stage | II | 0.541 | 0.307-0.954 | 0.0339 |
| VEGFA | stage | I | 1.646 | 1.01-2.684 | 0.0456 |
| VEGFA | stage | III | 0.533 | 0.295-0.963 | 0.0372 |
| CCL11 | stage | III | 0.503 | 0.279-0.905 | 0.022 |
| IL2 | T | T4 | 0.212 | 0.046-0.987 | 0.0481 |
| IL15 | T | T2 | 0.59 | 0.398-0.876 | 0.0088 |
| IL9 | T | T2 | 2.148 | 1.143-4.036 | 0.0175 |
| IL12B | T | T1 | 0.48 | 0.263-0.874 | 0.0163 |
| VEGFA | T | T4 | 0.135 | 0.041-0.449 | 0.0011 |
| IL12A | N | N2/N3 | 0.457 | 0.239-0.873 | 0.0176 |
| IL13 | N | N2/N3 | 0.465 | 0.231-0.936 | 0.0319 |
| IL12B | N | N0 | 0.622 | 0.406-0.953 | 0.029 |
| CCL11 | N | N2/N3 | 0.463 | 0.246-0.871 | 0.0168 |
| CCL5 | N | N0 | 0.637 | 0.414-0.979 | 0.0398 |
| IL2 | M | M0 | 0.639 | 0.449-0.909 | 0.0128 |
| CSF2 | M | M1 | 0.274 | 0.087-0.864 | 0.0272 |
| IL12B | M | M0 | 0.667 | 0.47-0.947 | 0.0235 |
| IL6 | M | M0 | 1.452 | 1.021-2.064 | 0.0378 |
| CSF3 | M | M0 | 1.591 | 1.12-2.262 | 0.0096 |
| IL10 | gender | Female | 0.584 | 0.388-0.88 | 0.0102 |
| IL12B | gender | Female | 0.546 | 0.364-0.818 | 0.0034 |
| IL12A | age | <=45 | 0.136 | 0.027-0.687 | 0.0157 |
| CXCL10 | age | <=45 | 0.093 | 0.018-0.466 | 0.0039 |
| IL15 | age | <=45 | 0.149 | 0.03-0.749 | 0.0208 |
| IL12B | age | >=65 | 0.555 | 0.371-0.831 | 0.0042 |
| CCL11 | age | <=45 | 0.123 | 0.024-0.617 | 0.0109 |
| CCL5 | age | <=45 | 0.075 | 0.009-0.617 | 0.016 |
| CCL4 | age | <=45 | 0.093 | 0.019-0.469 | 0.004 |
| CSF3 | age | <=45 | 0.221 | 0.052-0.935 | 0.0403 |
| IL2 | KRAS | KRAS WT | 0.64 | 0.455-0.899 | 0.0101 |
| CSF2 | KRAS | KRAS Mut | 0.54 | 0.301-0.968 | 0.0386 |
| IL12B | KRAS | KRAS WT | 0.621 | 0.441-0.872 | 0.006 |
| CXCL8 | EGFR | EGFR Mut | 2.585 | 1.001-6.677 | 0.0498 |
| IL4 | EGFR | EGFR Mut | 0.406 | 0.172-0.958 | 0.0395 |
| IL12B | EGFR | EGFR WT | 0.688 | 0.503-0.939 | 0.0186 |
| IL12B | EGFR | EGFR Mut | 0.318 | 0.132-0.769 | 0.011 |
| VEGFA | EGFR | EGFR Mut | 2.79 | 1.125-6.919 | 0.0268 |

**Supplemental Table 7.** Survival analysis result for RFS in LUAD patients with high and low cytokine expression under stratification of clinicopathological parameters.

| **Cytokine** | **Clinicopathological parameters** | **Group** | **Hazard Ratio** | **95% CI** | **Wald Test P** |
| --- | --- | --- | --- | --- | --- |
| CXCL8 | stage | I | 1.787 | 1.095-2.916 | 0.0202 |
| FGF2 | stage | II | 1.988 | 1.15-3.436 | 0.0138 |
| IL12B | stage | I | 0.502 | 0.306-0.822 | 0.0062 |
| VEGFA | stage | I | 1.85 | 1.131-3.024 | 0.0142 |
| IL6 | stage | II | 1.887 | 1.071-3.327 | 0.028 |
| IL10 | T | T3 | 2.883 | 1.113-7.467 | 0.0292 |
| IL12A | T | T1 | 2.731 | 1.29-5.778 | 0.0086 |
| IL15 | T | T3 | 3.116 | 1.205-8.053 | 0.019 |
| IL9 | T | T2 | 1.997 | 1.028-3.883 | 0.0413 |
| TNF | T | T3 | 3.673 | 1.443-9.351 | 0.0064 |
| VEGFA | T | T4 | 0.079 | 0.008-0.766 | 0.0285 |
| IL6 | T | T2 | 1.975 | 1.297-3.007 | 0.0015 |
| CCL3 | T | T3 | 3.005 | 1.126-8.021 | 0.0281 |
| CSF3 | T | T2 | 1.53 | 1.018-2.299 | 0.0408 |
| CXCL8 | N | N0 | 1.694 | 1.109-2.586 | 0.0146 |
| CXCL10 | N | N2/N3 | 0.385 | 0.169-0.881 | 0.0237 |
| FGF2 | N | N1 | 1.881 | 1.004-3.527 | 0.0487 |
| IL12B | N | N0 | 0.539 | 0.352-0.827 | 0.0046 |
| VEGFA | N | N0 | 1.632 | 1.069-2.491 | 0.0233 |
| IL6 | N | N0 | 1.538 | 1.005-2.354 | 0.0473 |
| IL6 | N | N1 | 1.942 | 1.03-3.663 | 0.0403 |
| CCL2 | N | N2/N3 | 0.395 | 0.181-0.864 | 0.0199 |
| CCL3 | N | N0 | 1.622 | 1.056-2.491 | 0.0272 |
| IL2 | M | M0 | 0.66 | 0.447-0.974 | 0.0364 |
| IL12B | M | M0 | 0.647 | 0.438-0.956 | 0.0286 |
| IL6 | M | M0 | 1.937 | 1.299-2.888 | 0.0012 |
| CSF3 | M | M0 | 1.993 | 1.338-2.969 | 7.00E-04 |
| IL10 | gender | Female | 0.612 | 0.403-0.928 | 0.0209 |
| IL1B | gender | Male | 1.659 | 1.009-2.726 | 0.0458 |
| CXCL8 | gender | Female | 1.68 | 1.101-2.564 | 0.0162 |
| IL12B | gender | Female | 0.584 | 0.386-0.884 | 0.0109 |
| VEGFA | gender | Female | 1.522 | 1.003-2.31 | 0.0484 |
| IL6 | gender | Female | 1.54 | 1.016-2.334 | 0.0417 |
| CCL3 | gender | Male | 1.764 | 1.071-2.907 | 0.0259 |
| CSF3 | gender | Male | 1.886 | 1.136-3.131 | 0.0142 |
| CXCL8 | age | 46-65 | 2.059 | 1.166-3.634 | 0.0128 |
| IL5 | age | 46-65 | 0.478 | 0.275-0.833 | 0.0091 |
| VEGFA | age | 46-65 | 1.879 | 1.057-3.339 | 0.0316 |
| CSF3 | age | >=65 | 1.666 | 1.09-2.547 | 0.0184 |
| CXCL8 | KRAS | KRAS WT | 1.473 | 1.02-2.127 | 0.0387 |
| IL12B | KRAS | KRAS WT | 0.638 | 0.442-0.921 | 0.0166 |
| IL6 | KRAS | KRAS WT | 1.539 | 1.066-2.221 | 0.0213 |
| CCL3 | KRAS | KRAS WT | 1.541 | 1.062-2.237 | 0.023 |
| IL4 | EGFR | EGFR Mut | 0.382 | 0.147-0.991 | 0.0479 |
| IL12B | EGFR | EGFR Mut | 0.36 | 0.137-0.947 | 0.0383 |
| VEGFA | EGFR | EGFR Mut | 4.96 | 1.616-15.222 | 0.0051 |
| IL6 | EGFR | EGFR WT | 1.595 | 1.134-2.244 | 0.0074 |
| CSF3 | EGFR | EGFR WT | 1.51 | 1.075-2.12 | 0.0174 |
